# Prevalence of camel babesiosis in southeast of Iran

**DOI:** 10.1002/vms3.666

**Published:** 2021-11-20

**Authors:** Hadi Mirahmadi, Ali Ghaderi, Shaghik Barani, Ebrahim Alijani, Ahmad Mehravaran, Reza Shafiei

**Affiliations:** ^1^ Infectious Disease and Tropical Medicine Research Center Research Institute of Cellular and Molecular Sciences in Infectious Diseases Zahedan University of Medical Sciences Zahedan Iran; ^2^ Department of Parasitology and Mycology Faculty of Medicine Zahedan University of Medical Sciences Zahedan Iran; ^3^ Department of Immunology School of Medicine Shiraz University of Medical Sciences Shiraz Iran; ^4^ Clinical Immunology Research Center Zahedan University of Medical Sciences Zahedan Iran; ^5^ Vector‐Borne Diseases Research Center North Khorasan University of Medical Sciences Bojnurd Iran

**Keywords:** babesiosis, camel, giemsa staining, microscopic examination, molecular technique, PCR

## Abstract

Babesiosis is a globally distributed zoonotic parasitic disease in a broad range of vertebrates with great importance in the veterinary field. The standard diagnostic test for Babesiosis in animals is microscopic identification of the parasite in a venous blood smear stained with Giemsa combined with assessment of clinical manifestations throughout the acute phase of the disease. The present study was planned to determine the presence of *Babesia* species in camels from the southeastern regions of Iran. A total of 140 blood samples of camels were randomly collected in four selected cities including Qaen, Nehbandan, Iranshahr, and Zahedan from March to August 2019. Blood smears of each case were also examined by the Giemsa staining method and extracted DNA samples were subjected to internal transcribed spacers (ITS1) polymerase chain reaction (PCR) amplification. The prevalence rates using microscopically and molecular examinations were 10% and 19.28%, respectively. The prevalence rates significantly vary between the selected regions (*p* = 0.003). PCR technique showed higher sensitivity than microscopy. We found that all infected camels were positive for *Babesia caballi*. The rate of infection with *Babesia* among the camel in Zahedan is remarkable. Early diagnosis and early treatment can prevent further spread of the disease in this area.

## Graphical abstract

Babesisosis in camels can be detected by microscopic and molecular diagnosis throughout the acute phase of disease. In the present study, Babesia species in camels from the eastern regions of the Iran was determined.

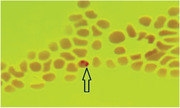

## 1 INTRODUCTION

Babesiosis is a globally distributed vector‐borne protozoan parasitic disease with considerable importance in veterinary and sometimes in medical professions (Haghi et al., 2014). The babesiosis causative agents are various species of the genus *Babesia* as apicomplexan intra‐erythrocytic parasites transmitted by several hard ticks of Ixodidae family belong to the genus *Hyaloma* spp., *Rhipicephalus* spp., and *Dermacentor* spp. to the variety of wild and domestic vertebrates, particularly to ruminants, dogs, cats, birds, rodents, and humans (Kalani et al. 2012). Several babesial species such as *Babesia microti, Babesia bovis, Babesia divergens, Babesia duncani*, and *Babesia venatorum* have been identified as causative agents for human babesiosis, which is associated with hemolytic anemia (Schnittger et al. 2012, Ord and Lobo 2015).

It shares all the morphological features of the apicomplexans. It is observed in various shapes including ring, oval, maltan cross, pear‐shaped, amoeboid form with a length of 1–2.5 or 2.5–5 μm within infected erythrocytes (Uilenberg, 2006). The standard diagnostic method for confirming babesiosis in animals is based on microscopic identification of parasites in a venous blood smear stained with Giemsa combined with assessment of clinical manifestations throughout the acute phase of the disease. During the career state of babesiosis, subclinical infections are identified in recovered animals. At the same time, sometimes no parasites are detectable by microscopic examination that can be the cause of false‐negative results (Terkawi et al., 2012). Since various parasite species can infect animals, the morphology‐based diagnosis of babesiosis is difficult (Bilgiç et al., 2013). Therefore, the application of molecular diagnostic tests can bring potential benefits such as more accurate identification of the *Babesia* species and differentiation from *Theilera* parasites, especially in the case of low parasitemia in a time and cost‐effective method (Alvarez et al. 2019; Bahrami et al. 2014; Motevalli Haghi et al. 2014). Molecular‐based techniques target internal transcribed spacers (ITS) among nuclear genomes located in the ribosomal region. ITS region is characterized as highly conserved sequences within species but variable between different species, so it is explicitly used as gene targets for identifying and discriminating different *Babesia* species (Liu et al., 2014). A majority of studies on the detection of babesiosis in infected animals have been performed on sheep and cattle in Iran, while there are limited studies on other species such as camels that could be infected by *Babesia* (Kalani et al., 2012; Sazmand & Joachim, 2017). Camel babesiosis with *B. caballi* as one the most important species of *Babesia* have been reported in a different part of the world (Abd‐Elmaleck et al., 2014; Ibrahim et al. 2017; Jasim et al., 2015; Khamesipour et al., 2015). During the acute stage of the disease, it causes fever, anemia, jaundice, and edema in the infected camels. It sometimes results in death which induces significant economic losses in the camel industry (Hosseini et al., 2017).

Camels are well known for surviving in harsh conditions in arid and semi‐arid regions and play an essential role in human life in Iran (Khalkhali‐Evrigh et al., 2018). Iranian camel population is around 150,000 in desert areas, particularly in Eastern regions of Iran. Ticks can transmit many different pathogenic organisms such as protozoan pathogens that cause piroplasmosis (Bitaraf Sani et al., 2020).

In the recent decade, consumption of camel dairy products and meat increased among Iranians, and therefore the control of infectious diseases is essential for food safety of camel products (Mohammadpour et al., 2020). Considering the inadequacy of data on the distribution of Babesiosis and genetic diversity of this parasite among camels in southeastern Iran, we aimed to investigate the prevalence and distribution of various *Babesia* species by polymerase chain reaction (PCR) method to address the research gap in this particular field.

## 2 MATERIALS AND METHODS

### 2.1 Study population and sampling

The present descriptive cross‐sectional study was conducted on 140 randomly included camels (*Camelus dromedarius*) in the spring and summer of 2019. Blood samples from the male (*N* = 86) and female (*N* = 54) camels brought for slaughter were gathered in Zahedan and Iranshahr from Sistan‐Baluchestan Province (southeastern Iran) and the Qaen and Nehbandan located in South Khorasan Province (Eastern Iran). The random sampling method was used to facilitate specimen collection. The sample size was determined according to previous research on other piroplasms and zoonotic parasites in Iran and studies on *Babesia*‐infected camels across the world (El‐Naga & Barghash, 2016; Sazmand & Joachim, 2017) by the following formula:

$$n=\frac{Z_{1-\alpha /2}P\left(1-P\right)}{d^{2}}$$

where *z* = 1.96, *p* = 0.1843, *d* = 0.057, and *α* = 0.05.

Blood samples were obtained from the jugular vein of camels in a sterile Venoject vacuum system and transferred to EDTA tubes. Following the preparation of thin fixed blood smears by a conventional method, few drops of blood were spotted on Whatman No. 4 filter papers and transferred to the parasitology laboratory of Zahedan University of Medical Sciences for molecular detection of different species of *Babesia*.

Blood smears were fixed in methanol then stained with Giemsa dye diluted with distilled water at the ratio 1:14 for 45 min. At least 50 microscopic fields per slide were examined at 1000× light microscope magnification for and morphological identification of *Babesia* piroplasms.

### 2.2 DNA extraction

According to the manufacturer's instructions, genomic DNA was extracted utilizing a Takapozist DNA extraction kit (Tehran, Iran). For each sample, 10 micro‐punches (1.2 mm) of blood‐spotted filter paper were placed into a 1.5‐ml microcentrifuge tube and centrifuged for 10 min at 12,000 rpm and the supernatant gently poured off. Two milliliters of 70% ethanol was added to the pellet, mixed by vortexing for 3–5 s, centrifuged at 12,000 rpm for 5 min, and the residual DNA was transferred into a new tube. Next, DNA concentration was determined in A260/A280 ratio by a NonoDrop spectrophotometer (Thermo Scientific, U.S.) and stored at –20°C until used.

### 2.3 Molecular detection of *Babesia* DNA

One‐step PCR targeting the ITS1 gene was performed to identify *Babesia* DNA in camels’ blood samples (Hilpertshauser et al., 2006; Khamesipour et al. 2015) using Bab‐sp‐F (GTTTCTGCCCCATCAGCTTGAC) as a forward primer and Bab‐sp‐R (CAAGACAAAAGTCTGCTTGAAAC) as a reverse primer (Cinnagen, Iran) (Ibrahim et al., 2017). Amplifications were performed in a total volume of 25 μl consisting of 2 μl genomic DNA, 0.5 μl of each primer (final concentration: 0.5 μM), 9.5 μl distilled water, and 12.5 μl of Taq 2× Master Mix (New England Biolabs, UK).

The amplification of *Babesia* spp. DNA was done using a Gene Amp 9700 thermal cycler (Applied Biosystems). The thermal program was as follows: initial incubation at 94°C for 30 s, followed by 45 cycles of 94°C for 20 s, 65°C for 30 s, and 68°C for 30 s, and a final extension at 72°C for 5 min.

Five microliters of the PCR amplification product with 1 μl loading dye (Qiagen, Germany) was mixed well and loaded into 1.5% agarose gel stained with ethidium bromide. Following DNA electrophoresis in TAE buffer, the gel documentation imaging system checked the agarose gel (Bio‐DocmM20).

### 2.4 Statistical analysis

Data analysis was carried out using SPSS software version 18 (IBM SPSS Statics 18, USA). Descriptive statistics, including frequency distribution, central and dispersion indexes together with chi‐square were applied for analysis and interpretation of research findings. All statistics were considered significant at *p*  < 0.05. Also, the kappa coefficient (*κ*) was used to measure agreement between the two screening diagnostic methods for babesiosis detection, indicating a relatively strong agreement between Giemsa staining microscopy and PCR methods.

## 3 RESULTS

**FIGURE 1:**
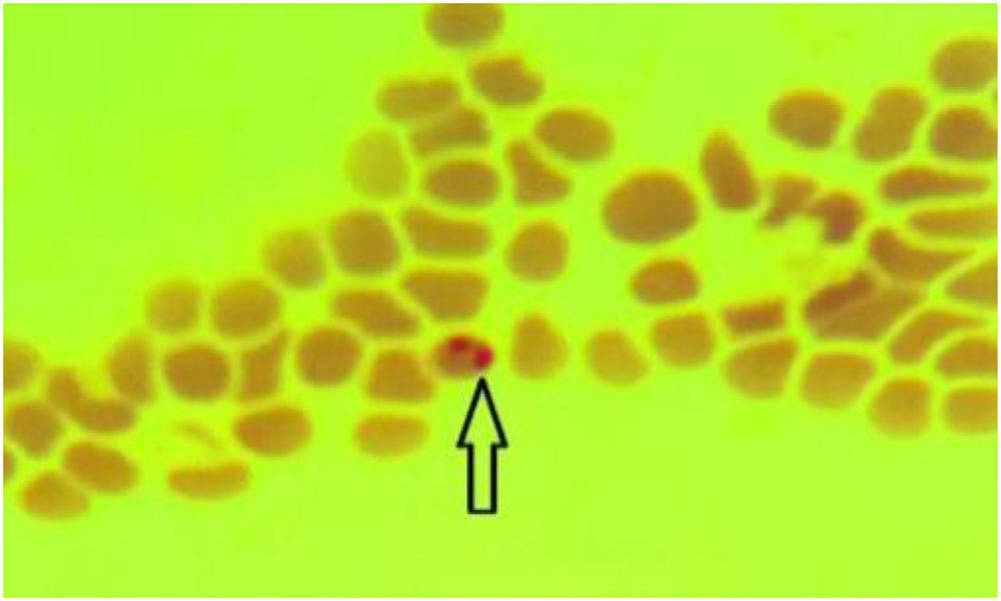
A sample of *Babesia* spp. in blood smear of camel in the east of Iran

**FIGURE 2:**
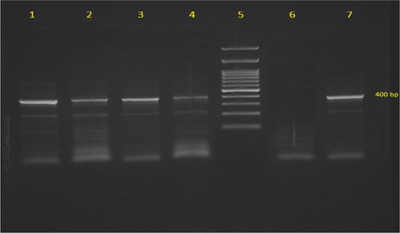
Agarose gel (1.5%) electrophoresis analysis of amplified DNA of *Babesia*. Lines 1–4: amplified 400 bp PCR product of *Babesia*. Line 5: 1000 bp DNA marker. Lines 6: negative control. Lines 7: positive control

By using the Giemsa staining method, *Babesia* infections were detected in 10% (*N* = 14) of camels (Figure 1) while a higher proportion (19.28%, *N *= 27) of infected camels was diagnosed by PCR (Table 1, Figure 2) (*p * > 0.05). DNA sequencing for identifying different species of *Babesia* revealed that all (100%) infected camels were positive for *Babesia caballi*.

**TABLE 1:** Measuring kappa coefficient between PCR and Giemsa‐stained microscopy test for detection of *Babesia* in camels

		PCR		
		Negative	Positive	Total
Giemsa staining for microscopy	Positive	0	14	14
	Negative	113	13	126
	Total	113	27	140
(κ)		0.635 (CI95%: 0.529–0.833)		

Kappa value is summarized in Table 2. As shown in Table 2, the kappa coefficient (*κ* = 0.635) showed an approximately strong relation between Giemsa staining microscopy and PCR screening.

**TABLE 2:** Comparison of PCR and Giemsa staining test in detecting infection with *Babesia* in camels across four cities in Iran

**City**	**Total number**	**Giemsa staining for microscopy**	***p*‐value**	**PCR**	***p*‐value**
**Iranshahr**	25	1 (4%)		1 (4%)	
**Qaen**	11	0 (0%)	0.29	1 (9.09%)	0.003
**Nehbandan**	40	3 (7.5%)		4 (10%)	
**Zahedan**	64	10 (15.6%)		21 (32.8%)	

**FIGURE 3:**
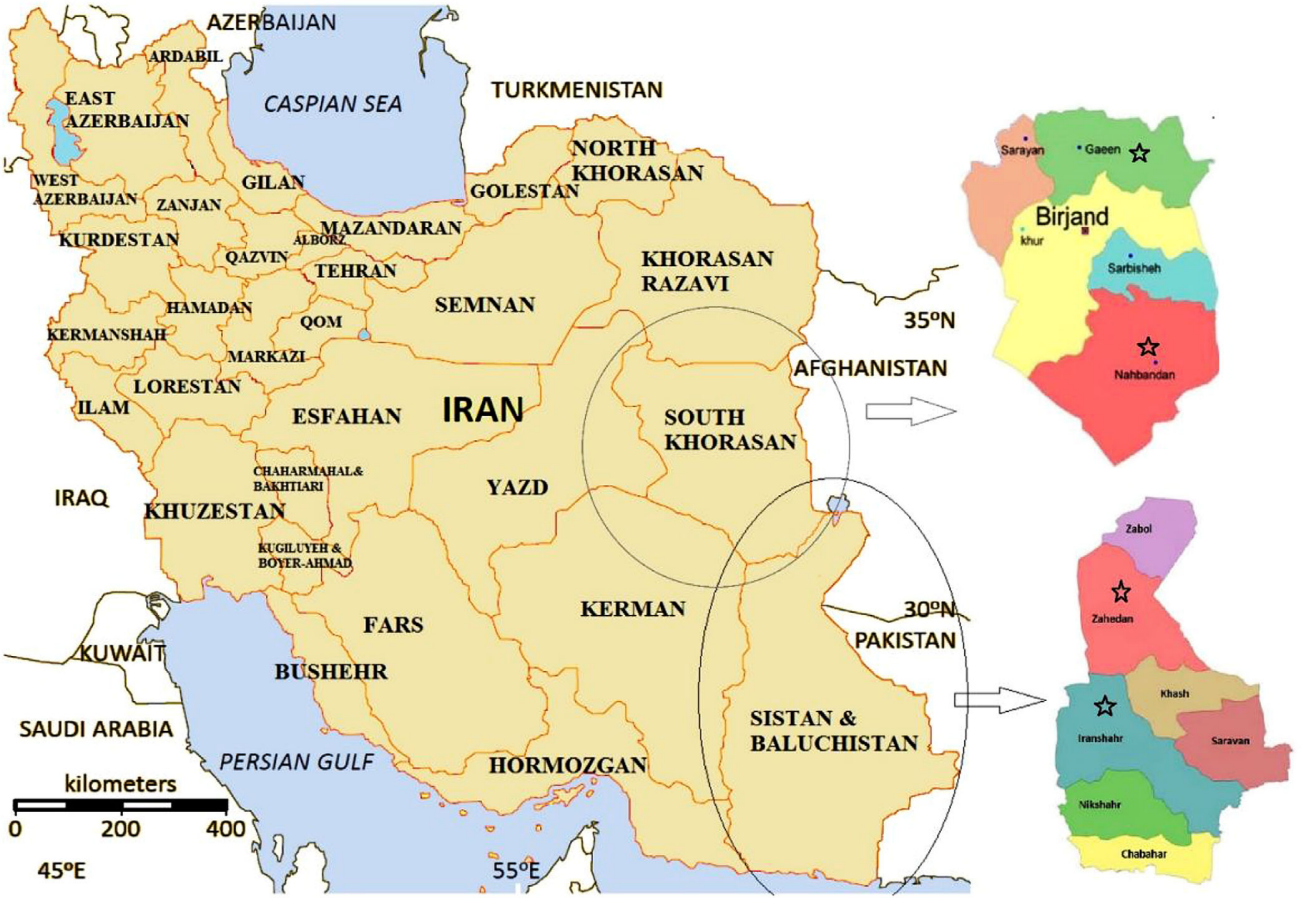
Prevalence and distribution patterns of babesiosis in camels of different cities (map from Google)

While reviewing the prevalence of *Babesia* infection in several cities of Sistan‐Baluchestan and South Khorasan Provinces as well, we observed a high prevalence rate of Babesiosis in camels across eastern regions of Iran (Figure 3) with the highest rate reported in Zahedan)*p* > 0.05) (Table 2). Table 2 shows that the prevalence of *Babesia* infection in camels of the studied cities using the Giemsa staining method was not significantly different (*p* < 0.05). At the same time, there were significant differences in prevalence rate when detected by PCR.

## 4 DISCUSSION

*Babesia* species as tick‐borne intraerythrocytic parasites are extensively distributed in tropical and subtropical regions of Iran, which can cause severe or even fatal diseases in several vertebrates during late spring and early summer (Rajabi et al., 2017). Camels are not considered a typical host for *Babesia* parasites. Hence, there is a lack of research on the *Babesia* infection in camels, particularly in Iran. However, some clinical manifestations of Babesiosis including fever, anemia, hemoglobinuria, jaundice, and various gastrointestinal disorders have been reported in infected camels (Swelum et al., 2014) and the transmission of B. caballi by infected ticks in Jordanian camels have been reported by Qablan and colleagues in 2012 (Qablan et al., 2012).

In the present study, among 140 camels examined for babesial infection, we identified 27 (19.29%) with *Babesia* infection when using PCR, while the detection rate was lower by Giemsa staining microscopy diagnosis with just 14 cases (10%). Considering the result of the study, a combination of microscopy and PCR‐based diagnostics is recommended. The result of our study confirmed the *Babesia* infection of camels in the study region of Iran. Our findings were in agreement with similar researches in Iran. In a study conducted on 2090 sheep slaughtered in Urmia city, 6.31% of samples were infected with *B. ovis* (Hajhoseynlo 1995) while in Ardabil city, a higher infection rate (44.9%) was observed in infected sheep from the same species. Moreover, 1.1% of the population was infected with *B. motasi* (Tavassoli & Haji‐Ghahremani, 2004). Razmi et al. (2003, 2002) reported the babesial infection rates of 25.1% and 14.5% in two different sample studies on sheep in Mashhad. In several studies on Babesiosis in small (sheep and goats) and large ruminants in Iran, the infection levels varied between 3.16% and 27.41% (Azizi et al., 2005; Noaman et al., 2005).

During the past decades, much more information has become available on Babesiosis in a wide variety of vertebrate hosts; while it has been addressed in several investigations in Iran, there have been few scientific investigations on *Babesia* infection in Iranian camels. In a study conducted in Zabol in 2008, the presence of *Babesia* was observed in 3.54% of camels detected by microscopic examination of blood smears (Ranjbar & Afshari, 2009). In another survey performed in Ahvaz, *Babesia* infections have been identified in eight out of 122 camels without determining the type of species (Khamesipour et al., 2015). Genome sequencing results revealed that the infection with *B. caballi* occurred in Iranian native camels with an infection rate of 1.2% in different regions of Iran in 2016–2017 (Bahrami et al. 2017; Ganjali Tafreshi, 2016).

Certain geographical and strategical specifications of the studied area and probably close communication of native camels with imported camels of neighboring countries including Afghanistan and Pakistan, and the distribution of vector ticks are factors that increase infection in this region. Therefore, this situation emphasizes the importance of border monitoring and quarantine, and control of vector ticks. However, research conducted on the prevalence of *Babesia* infection among camels was 5%–18.43% in Egypt (El‐Naga & Barghash, 2016; Mazyad & Khalaf, 2002; Nassar, 1992) and 74.5% in Sudan (Ibrahim et al., 2017). Comparison of the findings with previous studies reveals the increased prevalence of babesiosis in camels across the world and particularly in Iran over the past few years. A possible reason for the low prevalence of *B. caballi* could be associated with the earlier removal of the parasite after a short term of infection (Ibrahim et al., 2017). Hence, utilizing an appropriate diagnostic method for early detection of infection is required to break the chain of transmission among vertebrate hosts, which may avoid the spread of *Babesia* species in larger populations.

Although most of the infected cases remain asymptomatic, the clinical presentations of babesiosis are relatively similar to theileriosis. Therefore, generally, the detection of babesiosis is confirmed with the microscopic examination of blood smears.

Indeed, the most common and cost‐effective method for diagnosing babesiosis in vertebrates is a traditional microscopic examination of thin and thick blood smears obtained from superficial and deep vessels of live ruminants or heart, bone marrow, and kidney, or spleen regarding dead cases. However, it cannot be considered a reliable diagnostic method due to the issues like inaccurate identification of small‐sized *Babesia* species, even for experienced laboratory technicians (Motevalli Haghi et al., 2014). Our current findings demonstrated that the rate of *Babesia* diagnosis by PCR was almost twice the Giemsa staining method, which emphasizes the substantial role of PCR in detecting *Babesia* infection. Following our findings, El‐Naga and Barghash (2016) investigated the efficacy of the two mentioned methods in the diagnosis of *Babesia* and reported a greater rate of detection by PCR (18.43%) in comparison with the Giemsa staining (11.8%) method. Moreover, DNA sequencing of our samples has demonstrated that all detected cases with Babesiosis were infected by *B. caballi* which differs from the findings of Ibrahim et al. (2017), who detected just one infected case with *B. caballi* among 200 camels. Moreover, Jasim et al. (2015) stated that infection with *B. caballi* was identified in 39.47% and 31.57% of camels diagnosed by PCR utilizing two specific primers, thereby, camels should be considered as a source of *Babesia* infection since *B. caballi* can cause equine piroplasmosis that indicate the important role of camels in the epidemiology of babesiosis in horses. Finally, the preliminary analysis of Babesia's regional distribution among camels exhibited that the proportion of infected camels was remarkably higher in Zahedan compared with other selected cities except for Qaen.

Important factors include the easy movements of infected camels combined with animal trade over a large geographical area and the absence of isolation and separation procedures. In suspicious camels, lack of sufficient knowledge about acaricides and the high cost of vaccines can lead to increased incidence and spread of babesial infection among vertebrate hosts. Therefore, there is an urgent need to develop preventive measures in regions with the highest infection rate such as Zahedan to reduce the transmission of *Babesia*.

Taken together, this research has provided additional evidence concerning the efficacy of microscopic and molecular detection methods for *Babesia* spp. among camels in southeast regions of Iran. We want to emphasize the application of PCR as the most accurate and specific diagnostic method for *Babesia* infection, which can significantly benefit livestock farming practices by identifying *Babesia* coinfections, differentiation of *Babesia* species, and detection of low parasitic load which was negative in the microscopic inspection. Further experimental investigations are required to provide more detailed information about the geographical distribution of babesiosis, casual agents, and life cycle in various vertebrate hosts.

## AUTHOR CONTRIBUTIONS

All authors conceived and designed the study. AG conducted the sampling and experiments. EA and AM conducted the data analysis and interpretation. SB prepared the first draft of the manuscript. RS and HM supervised the study. All authors contributed to manuscript revision.

## CONFLICT OF INTEREST

We declare that all authors listed on the manuscript are employed by Iran's government as university researchers and faculty staff who do not have any primary function or representative other than research and/or education.

## ETHICAL APPROVAL

All the procedures in this investigation have been reviewed and approved by the Ethics Committee of Zahedan University of Medical Sciences (IR.Zaums.REC.1396.17).

### PEER REVIEW

The peer review history for this article is available at https://publons.com/publon/10.1002/vms3.666

## Data Availability

The data that support the findings of this study are available from the corresponding author upon reasonable request.
